# Inter-brain functional connectivity: Are we measuring the right thing?

**DOI:** 10.1371/journal.pone.0353371

**Published:** 2026-07-14

**Authors:** Juan Camilo Avendano-Diaz, Patrick Sothmann, Riitta Hari, Lauri Parkkonen

**Affiliations:** 1 Department of Neuroscience and Biomedical Engineering, Aalto University, Espoo, Finland; 2 Department of Art and Media, Aalto University, Espoo, Finland; Kochi University of Technology, JAPAN

## Abstract

Hyperscanning—the simultaneous recording of brain activity from multiple individuals—and the study of inter-brain synchronization is gaining popularity in social neuroscience. MEG/EEG hyperscanning studies often estimate inter-brain functional connectivity using phase-based metrics applied to oscillatory brain signals, assuming matching peak frequencies between the individuals studied. However, in reality peak frequencies typically differ between subjects and even between brain regions. Using simulated MEG/EEG signals, we systematically assessed how inter-individual frequency differences affect commonly used connectivity measures. Phase-based metrics were highly sensitive to frequency differences across individuals, whereas amplitude envelope correlation remained comparatively stable under these conditions. Our results underscore the need for connectivity metrics specifically tailored for inter-brain analyses. These findings are relevant to a range of disciplines that are increasingly integrating hyperscanning into their methodological toolkits.

## Introduction

**Fig 1:**
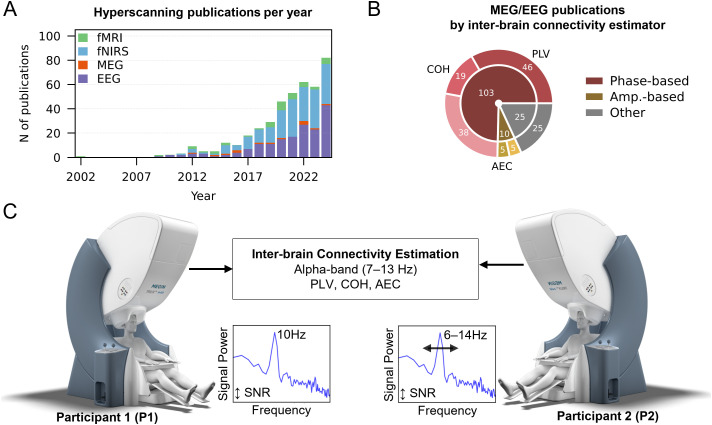
Hyperscanning landscape and simulation setup for evaluating inter-brain functional connectivity. (A) The number of hyperscanning publications per year, grouped by neuroimaging modality. (B) Magnetoencephalography (MEG) and electroencephalography (EEG) hyperscanning publications estimating inter-brain coupling, grouped by the type of connectivity metric employed (Phase-based, Amplitude-based, and Other approaches). The number of publications that use phase-locking value (PLV), coherence (COH), and amplitude-envelope correlation (AEC) is highlighted. The data were retrieved from Scopus using relevant search terms. (C) Schematic of our simulation framework. We generated MEG/EEG signals for two participants P1 and P2. The oscillatory signals P1 had a fixed frequency of 10 Hz. For P2, a set of oscillatory signals with varying frequency (6–14 Hz, 0.1-Hz steps) was created to simulate inter-individual variability in oscillatory peak frequencies. We estimated inter-brain functional connectivity in the alpha band (7–13 Hz), using PLV, COH, and AEC. Connectivity estimations were repeated while varying the signal-to-noise ratio (SNR) levels. MEG systems reprinted from megin.com under a CC BY license, with permission from Megin Oy, original copyright 2022.

Research on the brain basis of social interaction is complementing the traditional brain imaging studies with more naturalistic and interactive approaches. This methodological shift has been driven by calls to study two or more interacting individuals simultaneously, enabling insights that cannot be obtained through single-person paradigms [1–3]. As a result, hyperscanning, the simultaneous measurement of brain activity of two or more individuals [4], has been increasingly adopted across various noninvasive neuroimaging modalities, as reflected in the number of published hyperscanning studies during the last decade [5] (see Fig 1A).

Hyperscanning studies typically estimate interdependencies between the brain signals of interacting individuals using functional connectivity metrics [5]. The core assumption is that synchronization of brain activity between individuals, also referred to as inter-brain coupling, reflects aspects of social interaction [6]. However, most electroencephalography (EEG) and magnetoencephalography (MEG) hyperscanning studies rely on phase-based connectivity metrics originally developed to quantify frequency-specific synchronization between signals within a single brain [7] (see Fig 1B). These metrics assess dependencies between oscillations at equal frequencies. Yet, spontaneous neural oscillations vary in their peak frequencies between individuals, and within individuals across brain areas and tasks [8,9]. Even within certain brain regions, the sources and frequencies of the predominant oscillations can change dynamically depending on, e.g., vigilance and task. For instance, the sources of single spindles of the posterior alpha rhythm typically remain stable for less than a second [10]. Moreover, signal mixing across brain regions, an issue that affects more EEG than MEG data, can also contribute to inter-individual frequency differences. This variability is substantial [8,11–16]. In a sample of 51 healthy young adults, the peak frequency of the about 10-Hz posterior MEG alpha rhythm varied in different experimental conditions and brain regions (parietal and occipital) with an approximately three times larger between-subjects than within-subject variability (standard deviations 2.8 Hz and 0.9 Hz, respectively) [8]. Thus, variability is particularly relevant for hyperscanning datasets in which signals from different individuals are examined. Despite prior methodological discussions regarding the use of connectivity metrics in the hyperscanning field [17–19], the specific effect of inter-individual frequency mismatch on inter-brain connectivity estimates has not been systematically investigated. Because of the unaccounted inter-individual frequency differences, many MEG/EEG hyperscanning studies may have misestimated the functional interdependencies across interacting brains and the potential relation of the results to social interaction.

Here, we simulated oscillatory MEG/EEG signals for two participants (P1 and P2; see Fig 1C) to systematically investigate how inter-individual differences in oscillatory peak frequencies affect estimates of inter-brain functional connectivity. We computed connectivity in the 7–13 Hz “alpha band” using commonly employed phase-based metrics (see Fig 1B)—phase-locking value (PLV) and coherence (COH)—and evaluated amplitude envelope correlation (AEC) as an alternative. We also examined how signal-to-noise ratio (SNR) affects these estimates. Finally, we quantified the expected magnitude of inter-individual frequency mismatch in typical hyperscanning studies and illustrated its impact on the resulting inter-brain functional connectivity estimates.

## Materials and methods

We simulated MEG/EEG signals from two individuals (P1 and P2; see Fig 1C), systematically varying P2’s peak frequency (6–14 Hz, 0.1-Hz steps), while keeping P1’s frequency fixed at 10 Hz. We simulated these signals as the sum of three components: (1) an oscillation at the desired frequency, amplitude-modulated by an aperiodic 1/f^χ^ signal (0.005–0.5 Hz, χ = 0.5) in line with human physiology [20], (2) an aperiodic component with a pink noise exponent (χ = 1), and (3) white noise. For each scenario, we created with NeuroDSP [21] 50 trials of 30 s each, at a sampling rate of 1000 Hz. We estimated inter-brain functional connectivity between signals representing P1 and P2, in the alpha frequency band (7–13 Hz), using PLV, COH, and AEC. For PLV and COH, spectral decomposition was performed using complex Morlet wavelets with 3, 5, 7, and 10 cycles. AEC was obtained by applying the Hilbert transform to the bandpass-filtered signals to extract amplitude envelopes. Connectivity estimates were computed over time for each trial and then averaged across the 50 simulations. To manipulate SNR, we adjusted the amplitude of both P1 and P2’s signals while keeping the noise level constant, following the procedure used in Ref. [9]. We then assessed the effect of SNR on inter-brain connectivity using the connectivity estimation framework described above. We used MNE-Connectivity [22] for connectivity estimation.

To estimate the expected magnitude of inter-individual frequency mismatch in typical hyperscanning studies, we modeled the distribution of individual alpha peak frequencies (IAPF) based on empirical data. Specifically, we used the mean and standard deviation reported by Haegens et al. [8] for resting-state IAPF (μ=9.981 Hz, σ=1.088 Hz), and modeled IAPF as a normal distribution. This choise represents a conservative estimate of variability, as broader distributions have been reported in task-related data (e.g., μ=10.3 Hz, σ=2.8 Hz; [8]). We next computed the analytical distribution of pairwise (dyadic) frequency differences (Δf) between individuals independently sampled from the IAPF distribution. Under the assumption of independent sampling, Δf  follows a normal distribution with mean 0 and standard deviation $\sigma_{\Delta \text{f}}\;=\sqrt{\text{2}}\;\sigma$. Using this analytical distribution, we quantified the proportion of dyads expected to exhibit frequency mismatches exceeding predefined thresholds (|Δf|> 0.1 Hz and |Δf| > 1 Hz).

To illustrate the implications of the peak-frequency variability for inter-brain connectivity estimates, we additionally sampled N = 30 dyads (a sample size representative of hyperscanning studies) from the same resting-state IAPF distribution. For each sampled dyad, we generated oscillatory signals for both participants using sinusoidal components whose frequencies were independently drawn from this distribution and rounded to one decimal place. We then applied the same signal generation and inter-brain connectivity estimation framework described above. For each dyad, we computed PLV and AEC in the alpha band (7–13 Hz). This approach allows us to illustrate how empirically plausible inter-individual frequency variability translates into variability in inter-brain connectivity estimates across dyads.

## Results

**Fig 2:**
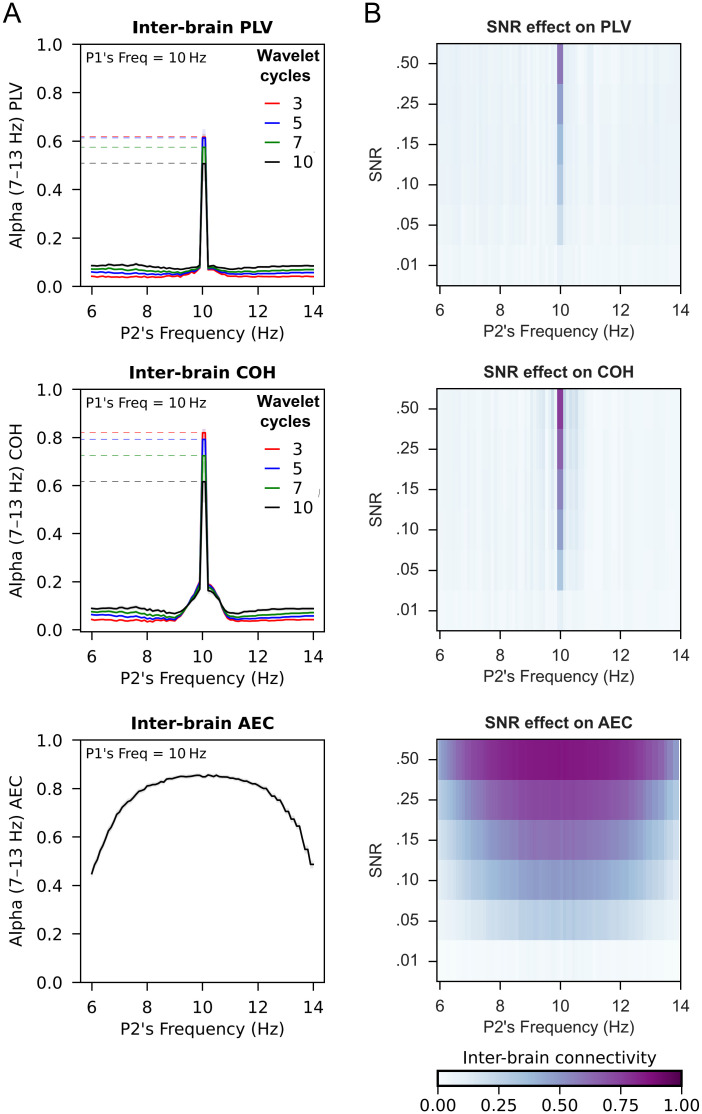
Effect of oscillatory peak frequency differences and signal-to-noise ratio on estimates of inter-brain functional connectivity. (A) Inter-brain connectivity between Participant 1 (P1; fixed frequency of 10 Hz) and Participant 2 (P2; 6–14 Hz, 0.1 Hz steps), estimated using PLV (top), COH (middle), and AEC (bottom) in the alpha band (7–13 Hz). The horizontal axis shows the peak frequencies of P2’s signals. Shaded areas represent 95% confidence intervals. (B) Inter-brain connectivity estimates across SNR levels. For PLV and COH, results are shown for 5 wavelet cycles; similar patterns were observed for 3, 7, and 10 cycles. Overall, phase-based connectivity estimates, unlike AEC, declined rapidly as P2’s frequency diverged from P1’s (10 Hz), regardless of the number of wavelet cycles employed.

Fig 2 summarizes the core simulation results. Estimates are referenced to the situation when P1 and P2 share a peak oscillatory frequency of 10 Hz. Here we refer to the accuracy of the connectivity estimates in terms of deviations from this reference value, which corresponds to the maximal connectivity estimate under the present simulation setup. We define robustness as the metric’s ability to maintain stable connectivity estimates under perturbations, such as inter-individual frequency differences or variations in signal-to-noise ratio.

### Phase-based inter-brain connectivity

PLV and COH were highly sensitive to inter-individual peak frequency differences (see Fig 2A) so that the accuracy of the of inter-brain connectivity estimates declined rapidly as the peak frequencies of P1 and P2 diverged. This effect was consistently observed across all applied numbers of wavelet cycles (3, 5, 7, and 10). Importantly, this effect was not limited to a specific choice of parameters or connectivity metric. Similar patterns were observed when varying the fixed peak frequency of P1 (7, 9, 11, and 13 Hz; see S1 Fig), and when using alternative phase-based connectivity measures, including phase lag index (PLI; [23]), weighted phase lag index (wPLI; [24]), corrected imaginary PLV (ciPLV; [25,26]), and maximised imaginary part of coherency (MIC; [25]) (see S2 Fig). Moreover, adjusting the frequency band definition to better align with individual alpha peaks did not mitigate the effect of frequency mismatch (see S3 Fig), nor did varying the epoch length within recommended ranges [18,27,28] (see S4 Fig).

### Amplitude-envelope-based inter-brain connectivity

In contrast to phase-based metrics, the AEC results were more robust to differences between the peak frequencies of P1 and P2 (see Fig 2A, bottom panel). The inter-brain connectivity estimates remained sensitive and stable across the frequency range of interest.

### SNR-dependence of inter-brain connectivity

Even when the peak oscillatory frequencies of P1 and P2 were equal (10 Hz), SNR reduction decreased connectivity estimates for all metrics (see Fig 2B). Notably, when the peak frequencies of P1 and P2 differed, the frequency mismatch dominated the results, overriding the influence of SNR.

### Impact of inter-individual frequency mismatch on hyperscanning studies

**Fig 3:**
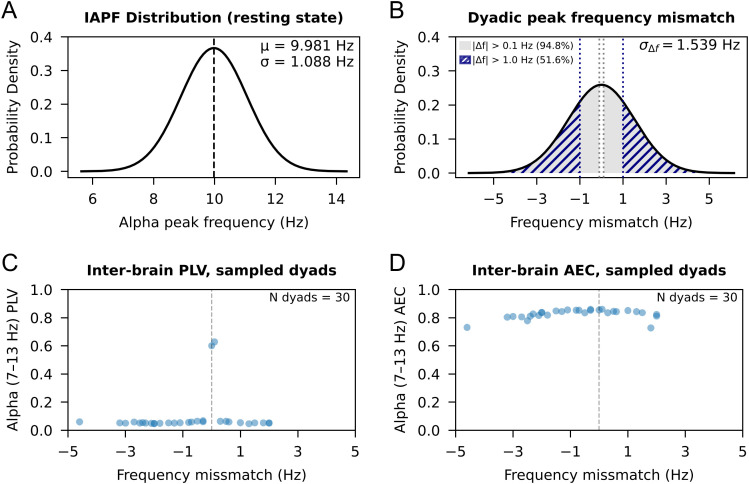
Population-level variability in alpha peak frequency and its impact on inter-brain connectivity estimates. (A) Analytical distribution of resting-state individual alpha peak frequencies (IAPF), modeled as a normal distribution (values taken from [8]). (B) Analytical distribution of pairwise frequency differences (Δf) between individuals independently sampled from the distribution shown in (A). Shaded regions indicate the proportion of dyads with frequency mismatches exceeding 0.1 Hz (94.8%) and 1 Hz (51.6%). (C) Inter-brain phase-locking value (PLV) estimated for N = 30 sampled dyads as a function of frequency mismatch. Individuals in each dyad were independently sampled from the distribution shown in (A). (D) Inter-brain amplitude envelope correlations (AEC) estimated for the same dyads. Blue dots represent individual dyads.

To assess the relevance of frequency mismatch under realistic conditions, we modeled the distribution of individual alpha peak frequencies (IAPF) based on empirical estimates and derived the corresponding distribution of pairwise differences (Fig 3A, 3B). The analytical results show that frequency mismatches are rather common. Approximately 94.8% of dyads are expected to differ by more than 0.1 Hz, and 51.6% by more than 1 Hz (Fig 3B). Notably, these values increase to 98% and 80.1%, respectively, when considering broader task-related variability (μ=10.3Hz, σ=2.8Hz; see [8]).

When sampling dyads from the resting-state IAPF distribution, inter-brain PLV estimates were strongly affected by frequency mismatch, with high values restricted to dyads with closely matched peak frequencies (Fig 3C). Notably, out of the 30 sampled dyads, only 2 showed relatively accurate inter-brain PLV values. In contrast, AEC estimates remained comparatively stable and accurate across the full range of observed mismatches (Fig 3D).

## Discussion

Our findings highlight a critical methodological limitation in the MEG/EEG hyperscanning literature: the phase-based connectivity metrics commonly used to examine inter-brain synchrony are highly sensitive to inter-individual differences in the peak frequency of spontaneous brain oscillations. Our simulations show that even small differences between the peak frequencies of participants in a hyperscanning study can lead to substantial misestimations of inter-brain connectivity based on this oscillatory activity (see Fig 2 and S2 Fig), which could compromise subsequent interpretations and potential links to social interaction.

Our results further show that the impact of inter-individual frequency mismatch on hyperscanning studies can be substantial. The empirically grounded population-level simulations (Fig 3) indicate that the magnitude of frequency mismatches typically present in the population is sufficient to affect phase-based inter-brain connectivity estimates in a large proportion of dyads, such that these estimates may not accurately reflect phase-based coupling across brains. Moreover, common mitigation strategies used in single-brain analyses, such as adjusting the frequency band definition to better align with individual alpha peaks (see S3 Fig) or varying the number of wavelet cycles (see Fig 2) did not mitigate the effect of frequency mismatch, nor did varying the epoch length within recommended ranges [18,27,28] (see S4 Fig.). Although the epoch-length-related results (S4 Fig) may suggest that short epoch lengths (e.g., 1 s) partially mitigate the effect of frequency mismatch, such choices are not recommended in practice. Previous work has shown that short epochs can lead to inflated and unreliable phase-based connectivity estimates, even in the absence of true coupling [18] and that connectivity estimates tend to stabilize only at longer epoch lengths (5–12 s; [18,27,28]). These observations suggest that the apparent improvement observed with shorter windows likely reflects other methodological biases rather than a genuine mitigation of the frequency mismatch effect.

Problems of using phase-based connectivity metrics in hyperscanning have been discussed earlier, demonstrating for example that PLV and COH are prone to detecting spurious couplings even in the absence of true inter-brain synchronization [17], and that common arbitrary methodological decisions can bias inter-brain coupling estimates [18]. Furthermore, in studies of infant–adult interactions, where large frequency mismatches are unavoidable, it has been acknowledged that employing conventional phase-based metrics may result in misleading interpretations [19]. Peak oscillatory frequencies have also been shown to vary across healthy aging [12], which calls for caution in intergenerational hyperscanning research. Alternative metrics that do not require strict frequency alignment, including cross-frequency coupling and symbolic mutual information, might help address these limitations [19]. However, the validity and interpretability of these approaches in hyperscanning still warrant systematic evaluation.

Whereas phase-based metrics misestimated phase coupling in the presence of peak frequency mismatches, AEC remained robust, providing relatively stable measures of amplitude coupling. This (expected) robustness makes AEC potentially more suitable for hyperscanning analyses. It is important to note that we simulated MEG/EEG signals from both individuals as continuous sinusoidal oscillations amplitude-modulated by an identical aperiodic signal, which is an idealized scenario for AEC that is unlikely to occur in real life. Although this setup is sufficient to demonstrate its robustness to frequency mismatch relative to phase-based metrics, we do not advocate for the use of AEC as a gold standard in hyperscanning based solely on the current results. A proper evaluation of AEC in hyperscanning contexts would require systematic benchmarking under explicit ground-truth conditions, including scenarios with varying levels of inter-brain coupling, common-driver effects simulating shared exogenous modulations, and different levels of correlated and independent noise across participants. Such benchmarking should also compare the performance of AEC against other connectivity metrics, including, for instance, cross-frequency coupling and information-theoretic approaches [29–34]. Moreover, AEC has its own limitations. It can be influenced by non-neural signal correlations, such as shared noise or stimulus-locked co-modulation, and typically requires longer data segments to yield accurate estimates [5,35]. AEC may also require additional steps, such as orthogonalization, to reduce zero-lag correlations arising from volume conduction in EEG or field spread in MEG [36], as well as the aforementioned instantaneous non-neural signal correlations [37]. As with any connectivity measure, its appropriateness depends on the specific research question, and the experimental context [35].

Our simulations further showed that the accuracy of inter-brain connectivity estimates was modulated by the SNR. These findings add to previous calls to carefully consider SNR differences between experimental conditions when interpreting results of intra- and inter-brain connectivity [9,18,35]. Our findings also suggest that the effect of inter-individual peak frequency differences has a dominant influence on the accuracy of inter-brain connectivity estimates compared to that of the SNR.

In sum, while hyperscanning offers a promising window into the brain basis of social interaction, it also presents unique methodological challenges [3]. Careful consideration of the limitations and validity of current connectivity metrics in inter-brain research is essential for obtaining valid and interpretable insights into how the brain activity of interacting individuals may support smooth social interaction. More broadly, our findings highlight the need to develop or adopt inter-brain connectivity metrics specifically tailored for hyperscanning datasets, and suggest that previous MEG/EEG hyperscanning findings should be interpreted with particular caution due to the sensitivity of commonly used metrics to frequency mismatch and signal-to-noise ratio.

Moving forward, we encourage the hyperscanning field to expand its methodological repertoire by developing and systematically comparing connectivity metrics that take human physiology into account. These additional tools include, for instance, brain physiological factors such as inter-individual variability in oscillatory properties, as well as cardiac, respiratory, and other visceral signals that can shape and coordinate neural dynamics [38–40] and therefore influence both intra- and inter-brain connectivity estimates. In this context, simultaneously measuring and analysing both brain and bodily signals may help clarify how peripheral physiological processes influence inter-brain connectivity measures [3,41]. To establish the validity and relevance of these functional connectivity metrics in hyperscanning, it is crucial to relate inter-brain coupling measures to behavioral, affective, psychological, and contextual factors of social interaction [3,37,42,43]. Recent developments, such as interaction-based phenotyping, which aim to derive observer-independent markers of how individuals relate to one another, may provide a promising complementary approach in this direction [42,44,45]. We also advocate for greater replication efforts and the joint analysis of intra- and inter-brain connectivity, which remain rare but could illuminate the unique contributions of shared and individual brain processes. Such steps are essential to ensure that hyperscanning continues to evolve into a rigorous approach for investigating the brain basis of real-world social interactions.

## Supporting information

S1 Fig Effect of oscillatory peak frequency differences on inter-brain functional connectivity across different reference frequencies. Inter-brain phase-locking value (PLV) as a function of P2’s oscillatory peak frequency (6–14 Hz), computed in the alpha band (7–13 Hz) using the simulation framework described in the Methods section. Here, P1’s peak frequency was set to 7 Hz (A), 9 Hz (B), 11 Hz (C), and 13 Hz (D). All other simulation parameters were identical to those used in the main analyses.
(TIF)

S2 Fig Effect of oscillatory peak frequency differences on inter-brain functional connectivity across phase-lag based metrics. Inter-brain connectivity as a function of P2’s oscillatory peak frequency (6–14 Hz), computed in the alpha band (7–13 Hz) using representative phase-lag-based metrics: (A) phase lag index (PLI), (B) weighted phase lag index (wPLI), (C) corrected imaginary phase-locking value (ciPLV), and (D) maximized imaginary part of coherency (MIC), as implemented in mne-connectivity. Simulations were identical to those used in the main analyses (P1 fixed at 10 Hz), with the addition of a phase offset between P1 and P2 (P2 delayed by a quarter cycle) to ensure non-zero phase differences. The sensitivity to oscillatory peak frequency mismatch is not limited to PLV and coherence, but generalizes to other phase-based metrics.
(TIF)

S3 Fig Effect of oscillatory peak frequency differences on inter-brain functional connectivity across different frequency band definitions. Inter-brain phase-locking value (PLV) as a function of P2’s oscillatory peak frequency (6–14 Hz), computed using different alpha band definitions centered on P1’s individual alpha frequency (IAF = 10 Hz): (A) IAF ± 2 Hz (8–12 Hz), (B) IAF ± 1 Hz (9–11 Hz), and (C) IAF ± 0.5 Hz (9.5–10.5 Hz). Simulations were otherwise identical to those used in the main analyses. Narrowing the frequency band around the IAF does not mitigate the effect of frequency mismatch on inter-brain connectivity estimates.
(TIF)

S4 Fig Effect of oscillatory peak frequency differences on inter-brain functional connectivity across different epoch lengths. Inter-brain phase-locking value (PLV) as a function of P2’s oscillatory peak frequency (6–14 Hz), computed in the alpha band (7–13 Hz) using different epoch lengths: (A) 1 s, (B) 3 s, (C) 5 s, (D) 7 s, and (E) 10 s. Simulations were otherwise identical to those used in the main analyses (P1 fixed at 10 Hz). The number of simulated epochs was adjusted across conditions to maintain a comparable total amount of data. Varying the epoch length within recommended ranges does not mitigate the effect of frequency mismatch on inter-brain connectivity estimates (see Discussion section). Short epoch lengths (e.g., 1 s) have been shown to lead to inflated and unreliable phase-based connectivity estimates, even in the absence of true coupling (see [18]).
(TIF)
