# Supplementary figures and images for: Inter-brain functional connectivity: Are we measuring the right thing?
Source: PLoS One. 2026 Jul 14;21(7):e0353371. doi: 10.1371/journal.pone.0353371 (PMC13367725; doi:10.1371/journal.pone.0353371)

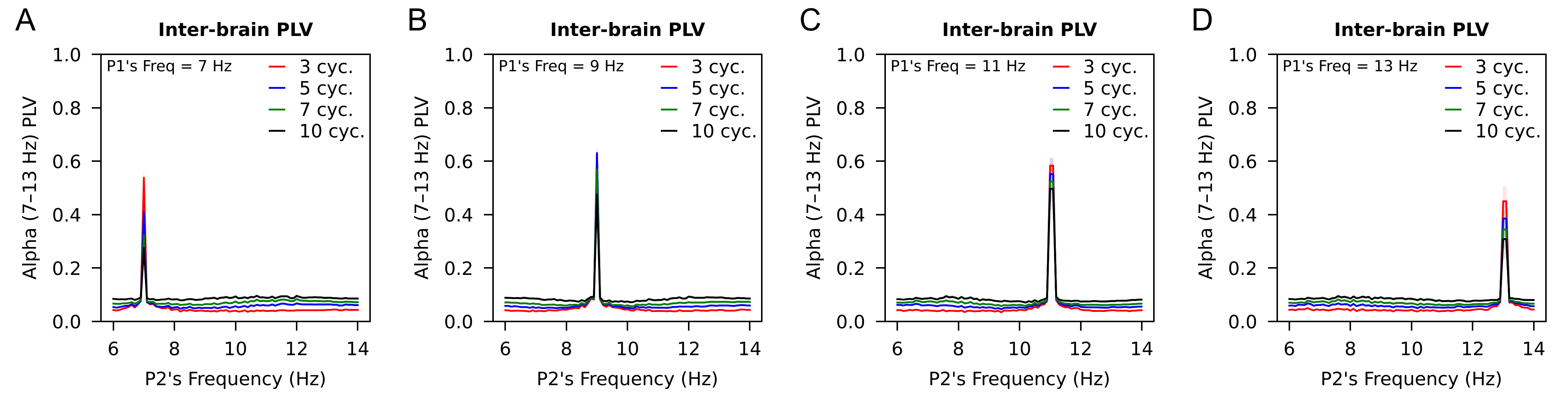

Supplement: S1 Fig — Inter-brain phase-locking value (PLV) as a function of P2’s oscillatory peak frequency (6–14 Hz), computed in the alpha band (7–13 Hz) using the simulation framework described in the Methods section. Here, P1’s peak frequency was set to 7 Hz (A), 9 Hz (B), 11 Hz (C), and 13 Hz (D). All other simulation parameters were identical to those used in the main analyses. (TIF) [file pone.0353371.s001.tif]

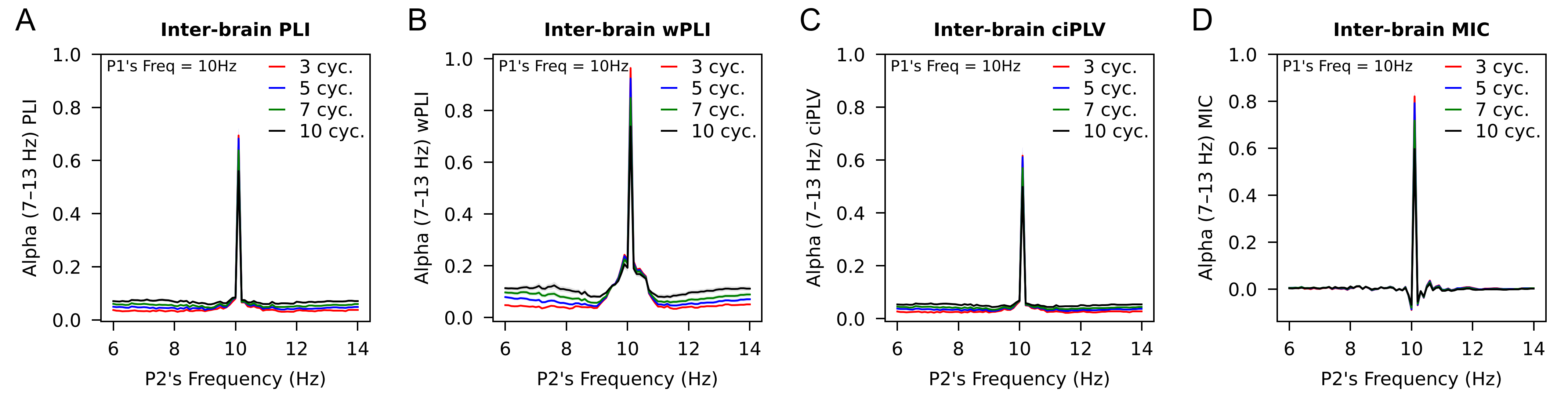

Supplement: S2 Fig — Inter-brain connectivity as a function of P2’s oscillatory peak frequency (6–14 Hz), computed in the alpha band (7–13 Hz) using representative phase-lag-based metrics: (A) phase lag index (PLI), (B) weighted phase lag index (wPLI), (C) corrected imaginary phase-locking value (ciPLV), and (D) maximized imaginary part of coherency (MIC), as implemented in mne-connectivity. Simulations were identical to those used in the main analyses (P1 fixed at 10 Hz), with the addition of a phase offset between P1 and P2 (P2 delayed by a quarter cycle) to ensure non-zero phase differences. The sensitivity to oscillatory peak frequency mismatch is not limited to PLV and coherence, but generalizes to other phase-based metrics. (TIF) [file pone.0353371.s002.tif]

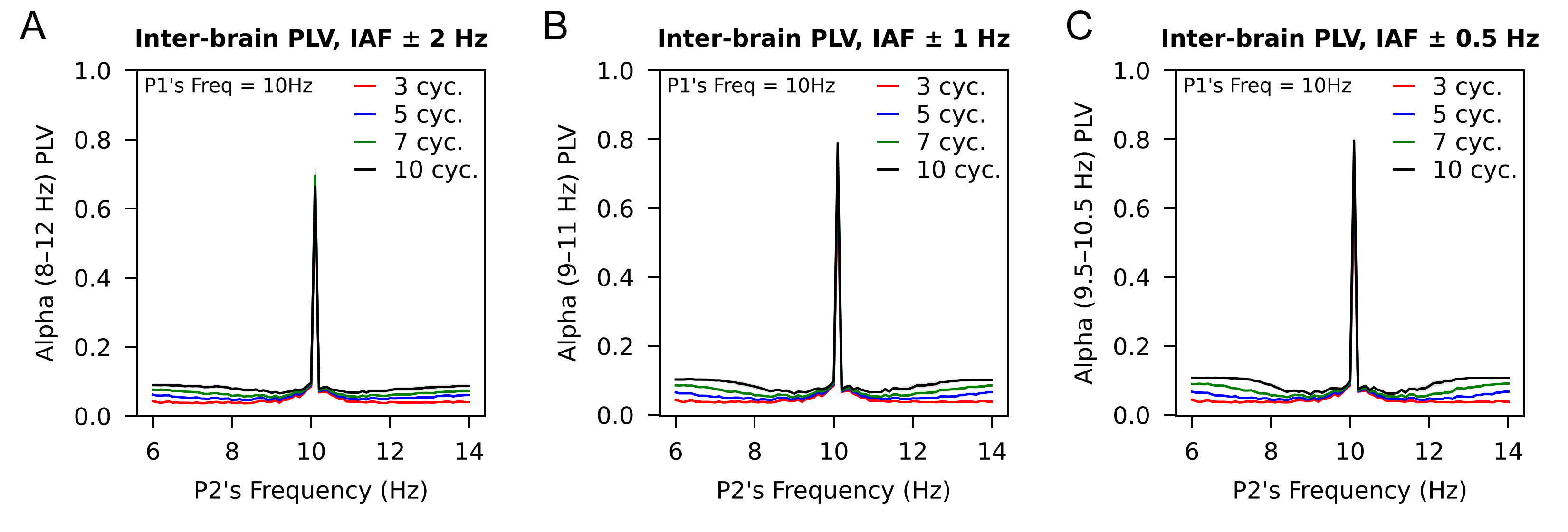

Supplement: S3 Fig — Inter-brain phase-locking value (PLV) as a function of P2’s oscillatory peak frequency (6–14 Hz), computed using different alpha band definitions centered on P1’s individual alpha frequency (IAF = 10 Hz): (A) IAF ± 2 Hz (8–12 Hz), (B) IAF ± 1 Hz (9–11 Hz), and (C) IAF ± 0.5 Hz (9.5–10.5 Hz). Simulations were otherwise identical to those used in the main analyses. Narrowing the frequency band around the IAF does not mitigate the effect of frequency mismatch on inter-brain connectivity estimates. (TIF) [file pone.0353371.s003.tif]

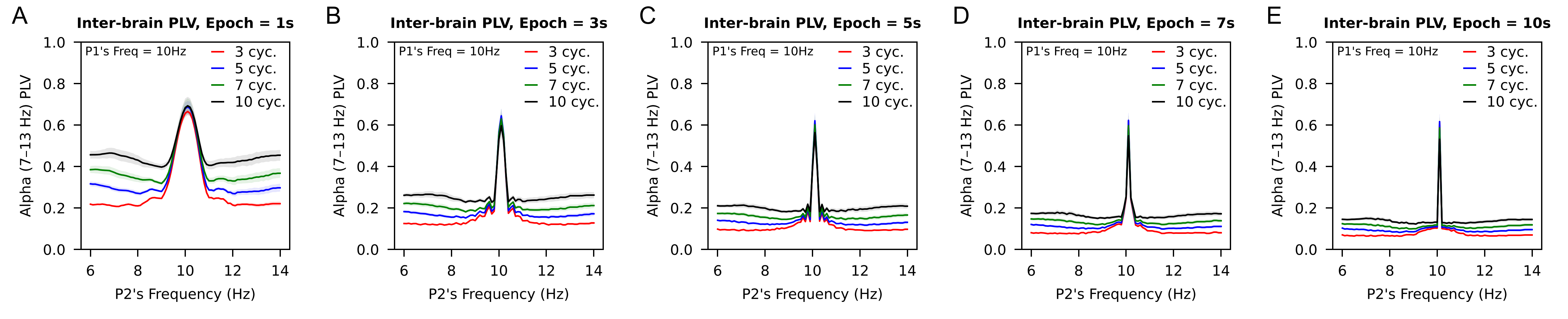

Supplement: S4 Fig — Inter-brain phase-locking value (PLV) as a function of P2’s oscillatory peak frequency (6–14 Hz), computed in the alpha band (7–13 Hz) using different epoch lengths: (A) 1 s, (B) 3 s, (C) 5 s, (D) 7 s, and (E) 10 s. Simulations were otherwise identical to those used in the main analyses (P1 fixed at 10 Hz). The number of simulated epochs was adjusted across conditions to maintain a comparable total amount of data. Varying the epoch length within recommended ranges does not mitigate the effect of frequency mismatch on inter-brain connectivity estimates (see Discussion section). Short epoch lengths (e.g., 1 s) have been shown to lead to inflated and unreliable phase-based connectivity estimates, even in the absence of true coupling (see [18]). (TIF) [file pone.0353371.s004.tif]
