# Supplementary material for: Inter-brain functional connectivity: Are we measuring the right thing?
Source: PLoS One. 2026 Jul 14;21(7):e0353371. doi: 10.1371/journal.pone.0353371 (PMC13367725; doi:10.1371/journal.pone.0353371)
Supplement: Attachment — Submitted filename: Response to Reviewers.pdf [file pone.0353371.s006.pdf]

Reviewer #1:

In this ms. the authors describe a much needed and timely study, in which they have used simulations of MEG and EEG data to highlight the need of developing novel and robust connectivity measures for use in inter-/cross-brain analyses of interacting dyads. They start out by reviewing the state of the art, the growing numbers of hyperscanning studies and group studies by the type of connectivity metric used. The authors cogently argue that previous studies have not been able to comprehensively account for inter-individual frequency differences (both across and within individual brains), which explains the need for methodological developments to address this issue. In their simulations they used oscillatory signals with a fixed for one interaction partner and with varying frequency for the other person, while employing various measures of cross-brain functional connectivity in the alpha band. These analyses were repeated for variable SNRs. Results demonstrate that phase-based interbrain connectivity measures were highly sensitive to inter-individual peak frequency differences. In contrast to this, amplitude-envelope-based measures of inter-brain connectivity were more robust to such differences and provide a more reliable measure of amplitude coupling. SNR reductions decreased connectivity estimates for all measures. I only have minor comments and strongly support the publication of this well-written ms. that is likely to make strong contribution to the relevant literature.

We appreciate Reviewer #1's positive evaluation of the manuscript and recognition of its relevance and contribution to the field, as well as the helpful suggestions for further clarification and contextualization.

-Would it not make sense to also use sets of oscillatory signals with varying frequency for both interaction partners? In recent years, the social interaction mismatch hypothesis as been formulated (e.g. Redcay & Schilbach 2019) and describes the idea that interpersonal similarity - at different levels of scientific assessment - could be related to communication success. Could the here described simulations be extended to address this?

We thank the reviewer for this question. We agree that the social interaction mismatch hypothesis (e.g., Redcay & Schilbach, 2019) provides a compelling framework, proposing that interpersonal similarity across multiple levels (so far primarily explored at behavioural and trait levels) may be linked to communication success, and that social impairments may arise from dynamic mismatches between interacting partners.

However, within the scope of inter-brain functional connectivity, our simulations suggest that the MEG/EEG hyperscanning field still requires further development and validation of metrics that can accurately capture such inter-brain alignment, before the social interaction mismatch hypothesis can be rigorously tested in terms of connectivity at the neural level. In addition to this, the social interaction mismatch hypothesis might operate at longer time scales than what is relevant for the frequencies of brain rhythms, although it may be relevant at the level of amplitude envelopes.

Extending our simulations to allow both participants' peak frequencies to vary would not alter the main conclusion of the manuscript. Rather, it would introduce a broader range of examples in which frequency mismatches lead to the same methodological limitation. To illustrate this, we included additional simulations (see S1 Fig.) in which the "fixed" frequency of P1 also varies across values (7, 9, 11, and 13 Hz), demonstrating that the observed effects generalize beyond the reference frequency (10 Hz) used in the main analyses.

We updated the results section to cite this supporting analysis:

“Importantly, this effect was not limited to a specific choice of parameters or connectivity metric. Similar patterns were observed when varying the fixed peak frequency of P1 (7, 9, 11, and 13 Hz; see S1 Fig)”

-The authors note that their MEG/EEG signal simulations as continuous sinusoidal oscillations modulated by an identical aperiodic signal that is unlikely to occur in real life, but do not go into any detail how this could be changed or addressed in future research. I would find this quite helpful.

We thank the reviewer for this comment, which we believe other readers may also share. We initially noted these limitations of our simulation framework to indicate that our results do not imply that AEC should be considered a gold standard for hyperscanning. Rather, we intended to highlight the need for future research to conduct thorough benchmarking studies, including AEC and other connectivity metrics.

We have now expanded this point in the Discussion with more explicit suggestions toward the future, in response to this comment and Reviewer 2’s Comment 2. Specifically, we added the following text:

“It is important to note that we simulated MEG/EEG signals from both individuals as continuous sinusoidal oscillations amplitude-modulated by an identical aperiodic signal, which is an idealized scenario for AEC that is unlikely to occur in real life. Although this setup is sufficient to demonstrate its robustness to frequency mismatch relative to phase-based metrics, we do not advocate for the use of AEC as a gold standard in hyperscanning based solely on the current results. A proper evaluation of AEC in hyperscanning contexts would require systematic benchmarking under explicit ground-truth conditions, including scenarios with varying levels of inter-brain coupling, common-driver effects simulating shared exogenous modulations, and different levels of correlated and independent noise across participants. Such benchmarking should also compare the performance of AEC against other connectivity metrics, including, for instance, cross-frequency coupling and information-theoretic approaches [29–34]”

-In the discussion the authors suggest that connectivity metrics should take human physiology into account, but leave open how this could be done. Are thinking more in terms of brain physiology or would they also consider whole-body physiology, as for instance suggested by the growing field of brain-heart-introspection science?

We thank the reviewer for this important point. In the manuscript, our reference to “taking human physiology into account” primarily relates to brain physiology, for instance, inter-individual variability in oscillatory properties, which directly impact the estimation of inter-brain connectivity in MEG/EEG.

At the same time, we agree that whole-body physiology represents an important and emerging dimension for understanding brain function, brain–body interactions, and social interaction. Growing evidence suggests that peripheral physiological processes can shape neural dynamics, and may therefore also influence inter-brain connectivity estimates.

We have revised the manuscript accordingly, adding the following text to the discussion section:

“These additional tools include, for instance, brain physiological factors such as inter-individual variability in oscillatory properties, as well as cardiac, respiratory, and other visceral signals that can shape and coordinate neural dynamics [38–40] and therefore influence both intra- and inter-brain connectivity estimates. In this context, simultaneously measuring and analysing both brain and

bodily signals may help clarify how peripheral physiological processes influence inter-brain connectivity measures [3,41]"

-The authors also stress that cross-brain connectivity measures should be related to social behavior, but also do not touch upon recent developments towards observer-dependent measures of social interaction (e.g. Lahnakoski et al. 2020 and others) that might be helpful along the way.

We thank the reviewer for this suggestion. We agree that observer-independent measures of social interaction represent an important complementary approach toward relating inter-brain connectivity measures to social behaviour.

We revised the manuscript accordingly, adding the following text to the discussion section:

To establish the validity and relevance of these functional connectivity metrics in hyperscanning, it is crucial to relate inter-brain coupling measures to behavioral, affective, psychological, and contextual factors of social interaction [3,37,42,43]. Recent developments, such as interaction-based phenotyping, which aim to derive observer-independent markers of how individuals relate to one another, may provide a promising complementary approach in this direction [42,44,45].

Reviewer #2:

This manuscript uses simulations to examine a well-known property of same-frequency phase-based synchrony metrics in the specific context of MEG/EEG hyperscanning: when two signals have different dominant (peak) frequencies, phase-based measures computed within a fixed band can rapidly lose apparent synchrony. In the presented simulations, PLV and coherence decrease as the peak frequency of participant 2 diverges from participant 1, while amplitude envelope correlation (AEC) remains relatively stable. The question is relevant to hyperscanning practice because inter-individual peak-frequency variability is common, and many studies still apply within-brain connectivity measures to across-brain data without explicit frequency alignment.
However, several aspects of the simulation design and the framing of conclusions make the paper's claims broader than what is demonstrated. In particular, the simulations appear to favour AEC by construction (shared envelope modulator across participants) and do not establish "ground truth" coupling conditions needed to support strong claims about reliability or about the extent of misestimation in the literature. In addition, novelty and positioning relative to prior methodological discussions are unclear.

We appreciate Reviewer #2's careful and critical assessment, which has helped us improve the conceptual clarity, methodological framing, and positioning of the work, making the revised version of the manuscript more informative to the reader.

Major comments

1. The central effect demonstrated—that phase synchronization metrics at a given frequency degrade when the compared signals are at different frequencies—is a general property of same-frequency phase metrics, not something unique to inter-brain analyses. The manuscript frames the issue as a "critical limitation" of hyperscanning work, but the novelty is currently unclear unless the authors show what is specifically different (or more severe) in hyperscanning practice versus within-brain connectivity (e.g., typical cross-person mismatch distributions, developmental dyads, region-specific peak differences, time-varying peak frequency, etc.). As written, the work

risks restating a general signal-processing property without sufficiently demonstrating new insight specific to hyperscanning.

We thank the reviewer for this very relevant comment. We, of course, fully agree that the sensitivity of same-frequency phase-based metrics to frequency mismatch is a general signal-processing property. Our motivation for this manuscript was that this property has been largely overlooked in hyperscanning research. To better clarify the novelty and relevance of our work in this context, we have revised the Introduction to more explicitly reflect the magnitude of inter-individual variability and to better position our study within the existing literature.

We included the following text in the Introduction:

"This variability is substantial [8,11–16]. In a sample of 51 healthy young adults, the peak frequency of the about 10-Hz posterior MEG alpha rhythm varied in different experimental conditions and brain regions (parietal and occipital) with an approximately three times larger between-subjects than within-subject variability (standard deviations 2.8 Hz and 0.9 Hz, respectively) [8]. Thus, variability is particularly relevant for hyperscanning datasets in which signals from different individuals are examined. Despite prior methodological discussions regarding the use of connectivity metrics in the hyperscanning field [17–19], the specific effect of inter-individual frequency mismatch on inter-brain connectivity estimates has not been systematically investigated.

2. Based on the Methods, P1 and P2 signals appear to be generated independently except for sharing an identical slow aperiodic modulator (the oscillation is "amplitude-modulated by an identical aperiodic 1/fχ signal"). This design choice is important: it makes amplitude envelopes correlated across participants by construction, which will favour AEC even when carrier frequencies differ. The Discussion acknowledges that the AEC scenario is idealized, but the main take-home message still implies AEC is broadly "more reliable."
To support claims about "reliable connectivity estimates," the simulations should include explicit ground-truth conditions that separate:
- **true inter-brain phase coupling (present vs absent),**
- **true inter-brain amplitude coupling (present vs absent),**
- **common-driver scenarios (shared exogenous modulation), and**
- **correlated noise across participants versus independent noise.**

Without these controls, the current AEC result is not an informative benchmark for real hyperscanning datasets, where stimulus-locked co-modulation and shared environmental noise are common confounds.

We thank the reviewer for raising this important concern. We agree that the current AEC results do not constitute an informative benchmark for hyperscanning applications, and that, at this stage, we cannot make claims about the reliability of AEC. The primary aim of this manuscript is to raise the question of whether commonly used approaches are adequately capturing inter-brain functional connectivity, particularly in light of the often overlooked inter-individual frequency mismatches. A thorough evaluation of which metrics are most appropriate for hyperscanning will require systematic benchmarking across multiple connectivity measures, including AEC, and is beyond the scope of the present study.

We have revised the manuscript accordingly to clarify this and to avoid general claims about the reliability of AEC. Specifically, the discussion section now states:

"It is important to note that we simulated MEG/EEG signals from both individuals as continuous sinusoidal oscillations amplitude-modulated by an identical aperiodic signal, which is an idealized

scenario for AEC that is unlikely to occur in real life. Although this setup is sufficient to demonstrate its robustness to frequency mismatch relative to phase-based metrics, we do not advocate for the use of AEC as a gold standard in hyperscanning based solely on the current results. A proper evaluation of AEC in hyperscanning contexts would require systematic benchmarking under explicit ground-truth conditions, including scenarios with varying levels of inter-brain coupling, common-driver effects simulating shared exogenous modulations, and different levels of correlated and independent noise across participants. Such benchmarking should also compare the performance of AEC against other connectivity metrics, including, for instance, cross-frequency coupling and information-theoretic approaches [29–34]

3. Only PLV and "coherence" are used as phase-based measures. Hyperscanning papers commonly use phase-lag metrics aimed at mitigating spurious zero-lag coupling and mixing artefacts (e.g., imaginary coherence, wPLI). Even if these metrics are still sensitive to frequency mismatch, readers will expect them to be considered or clearly explained as out of scope. At minimum, the authors should either (a) include representative phase-lag metrics to show that mismatch sensitivity generalises, or (b) explicitly restrict the scope to frequency-mismatch effects and avoid general claims about "phase-based metrics" in hyperscanning.
Similarly, AEC is often computed with additional steps (e.g., orthogonalization) in EEG/MEG connectivity work to reduce leakage-driven correlations. Because the manuscript raises signal mixing as relevant (especially for EEG), the choice of AEC implementation and its implications should be discussed and/or tested.

We thank the reviewer for this suggestion. We employed PLV and coherence as representative phase-based metrics because, according to our survey of the literature, they are among the most commonly used inter-brain connectivity measures in MEG/EEG hyperscanning studies.

However, we agree that explicitly demonstrating that the sensitivity to frequency mismatch generalizes beyond these metrics strengthens the manuscript and provides a more complete picture for the reader. To address this, we extended our analyses to include representative phase-lag-based measures, including PLI, wPLI, ciPLV, and MIC (see S2 Fig.), as implemented in mne-connectivity. We used the same simulation parameters as in the original analyses, with the addition of a phase offset between P1 and P2 (i.e., P2's signals delayed by a quarter cycle) to ensure non-zero phase differences, as required for phase-lag-based metrics to yield non-zero values.

As shown in the revised results, connectivity estimates obtained with these phase-lag metrics also remain strongly dependent on frequency alignment. This demonstrates that sensitivity to frequency mismatch is not limited to PLV and coherence, but generalizes to other phase-based metrics. We included this in the results section of the manuscript: "Importantly, this effect was not limited to a specific choice of parameters or connectivity metric. Similar patterns were observed when varying the fixed peak frequency of P1 (7, 9, 11, and 13 Hz; see S1 Fig.), and when using alternative phase-based connectivity measures, including phase lag index (PLI; [23]), weighted phase lag index (wPLI; [24]), corrected imaginary PLV (ciPLV; [25,26]), and maximised imaginary part of coherency (MIC; [25]) (see S2 Fig.). "

Regarding AEC, we also revised the manuscript to include an explicit mention to "orthogonalization" as an additional choice to consider:

"For instance, it can be influenced by non-neural signal correlations, such as shared noise or stimulus-locked co-modulation, and typically requires longer data segments to yield accurate estimates [5,35]. AEC may also require additional steps, such as orthogonalization, to reduce zero-

lag correlations arising from volume conduction in EEG or field spread in MEG [36], as well as the aforementioned instantaneous non-neural signal correlations [22]."

4. Band definition and common alignment strategies are not tested
The simulation computes connectivity in a fixed alpha band (7–13 Hz) while P1 is fixed at 10 Hz and P2 varies 6–14 Hz. Many analysis pipelines instead use individual alpha frequency (IAF)-centred bands or compute connectivity at subject-specific peaks. If the practical recommendation is that researchers should not assume identical peak structure across brains, the paper would be more actionable if it tested at least one standard mitigation strategy (e.g., IAF±2 Hz per person, or connectivity computed at each participant's own peak with an explicit cross-person mapping). As presented, the simulations quantify the expected failure mode of fixed-band, same-frequency phase metrics under mismatch—but do not show how much standard practices mitigate the issue.

We thank the reviewer for this suggestion. We agree that individual alpha frequency (IAF) -centred approaches are commonly used to mitigate inter-individual variability in oscillatory peak frequencies in single-brain analyses, and that readers may find it informative to assess whether such strategies can address the peak frequency mismatch issue highlighted in our manuscript.

To address this, we extended our simulations (see S3 Fig.) by computing connectivity using different alpha band definitions that approximate the commonly used IAF-based approaches. Specifically, simulations were performed assuming an IAF of 10 Hz for P1, consistent with the core example presented in the manuscript. We evaluated connectivity in the frequency bands 7–13 Hz (canonical alpha band), 8–12 Hz (approximating IAF ± 2 Hz), 9–11 Hz (IAF ± 1 Hz), and 9.5–10.5 Hz (IAF ± 0.5 Hz). Narrowing the frequency band around the IAF of one participant does not mitigate the effect of peak frequency mismatch on inter-brain connectivity estimates.

Computing connectivity at each participant's individual peak frequency (when they differ) is not directly compatible with classic phase-based connectivity metrics, which are inherently defined at matched frequencies across signals. We note, however, that alternative approaches (such as cross-frequency coupling metrics) may provide a way to relate signals at different frequencies across individuals. As discussed in the manuscript, the validity and interpretability of such approaches in hyperscanning contexts require systematic evaluation in future benchmarking studies.

To make the manuscript more informative to the reader, we also extended our analysis to examine the effect of using different epoch lengths for connectivity estimation (see S4 Fig). We revised the manuscript to discuss the relevant results.

We updated the results section accordingly, citing the relevant supporting information:

"Moreover, adjusting the frequency band definition to better align with individual alpha peaks did not mitigate the effect of frequency mismatch (see S3 Fig.), nor did varying the epoch length within recommended ranges [18,27,28] (see S4 Fig.). "

We also added the following to the discussion section:

"Moreover, common mitigation strategies used in single-brain analyses, such as adjusting the frequency band definition to better align with individual alpha peaks (see S3 Fig.) or varying the number of wavelet cycles (see Fig. 2) did not mitigate the effect of frequency mismatch, nor did varying the epoch length within recommended ranges [18,27,28] (see S4 Fig.). Although the epoch-length-related results (S4 Fig.) may suggest that short epoch lengths (e.g., 1 s) partially mitigate the effect of frequency mismatch, such choices are not recommended in practice. Previous work has shown that short epochs can lead to inflated and unreliable phase-based connectivity estimates,

even in the absence of true coupling [18] and that connectivity estimates tend to stabilize only at longer epoch lengths (5–12 s; [18,27,28]). These observations suggest that the apparent improvement observed with shorter windows likely reflects other methodological biases rather than a genuine mitigation of the frequency mismatch effect."

5. Claims about the literature are stronger than the evidence provided
The manuscript suggests that many hyperscanning studies "may have misestimated" inter-brain interdependencies due to unaccounted frequency differences. This is plausible, but currently speculative. The paper does not provide evidence about typical mismatch magnitudes in real hyperscanning datasets, nor does it quantify how often common pipelines would produce meaningful underestimation or false negatives. A more defensible statement, based on the simulations shown, would be: phase-based measures computed within a fixed band can strongly underestimate coupling when peak frequencies differ; therefore, studies assuming aligned peaks without testing alignment risk underestimating inter-brain coupling and producing inconsistent results across samples.

We thank the reviewer for this very relevant comment. We agree that, in the original version of the manuscript, statements regarding the potential impact of frequency mismatch on the hyperscanning literature might sound rather speculative considering that the manuscript didn't provide explicit values that would allow an idea of the typical mismatch magnitudes in the general population or the impact on hyperscanning studies.

To address this, we extended our analyses to better ground our claims in empirically informed estimates of inter-individual variability.

More specifically, we added the following analyses to the methods section:

"To estimate the expected magnitude of inter-individual frequency mismatch in typical hyperscanning studies, we modeled the distribution of individual alpha peak frequencies (IAPF) based on empirical data. Specifically, we used the mean and standard deviation reported by Haegens et al. [8] for resting-state IAPF ($\mu = 9.981\ Hz, \sigma = 1.088\ Hz$), and modeled IAPF as a normal distribution. This choise represents a conservative estimate of variability, as broader distributions have been reported in task-related data (e.g., $\mu = 10.3$ Hz, $\sigma = 2.8$ Hz; [8]). We next computed the analytical distribution of pairwise (dyadic) frequency differences (Δf) between individuals independently sampled from the IAPF distribution. Under the assumption of independent sampling, Δf follows a normal distribution with mean 0 and standard deviation $\sigma_{\Delta f} = \sqrt{2}\,\sigma$. Using this analytical distribution, we quantified the proportion of dyads expected to exhibit frequency mismatches exceeding predefined thresholds ($|\Delta f| > 0.1$ Hz and $|\Delta f| > 1$ Hz).

To illustrate the implications of the peak-frequency variability for inter-brain connectivity estimates, we additionally sampled N = 30 dyads (a sample size representative of hyperscanning studies) from the same resting-state IAPF distribution. For each sampled dyad, we generated oscillatory signals for both participants using sinusoidal components whose frequencies were independently drawn from this distribution and rounded to one decimal place. We then applied the same signal generation and inter-brain connectivity estimation framework described above. For each dyad, we computed PLV and AEC in the alpha band (7–13 Hz). This approach allows us to illustrate how empirically plausible inter-individual frequency variability translates into variability in inter-brain connectivity estimates across dyads."

We updated the results section:

"Impact of inter-individual frequency mismatch on hyperscanning studies

To assess the relevance of frequency mismatch under realistic conditions, we modeled the distribution of individual alpha peak frequencies (IAPF) based on empirical estimates and derived the corresponding distribution of pairwise differences (Fig. 3A,B). The analytical results show that frequency mismatches are rather common. Approximately 94.8% of dyads are expected to differ by more than 0.1 Hz, and 51.6% by more than 1 Hz (Fig. 3B). Notably, these values increase to 98% and 80.1%, respectively, when considering broader task-related variability ($\mu = 10.3$ Hz, $\sigma = 2.8$ Hz; see [8]).

When sampling dyads from the resting-state IAPF distribution, inter-brain PLV estimates were strongly affected by frequency mismatch, with high values restricted to dyads with closely matched peak frequencies (Fig. 3C). Notably, out of the 30 sampled dyads, only 2 showed relatively accurate inter-brain PLV values. In contrast, AEC estimates remained comparatively stable and accurate across the full range of observed mismatches (Fig. 3D)."

We also updated the discussion section:

"Our results further show that the impact of inter-individual frequency mismatch on hyperscanning studies can be substantial. The empirically grounded population-level simulations (Fig. 3) indicate that the magnitude of frequency mismatches typically present in the population is sufficient to affect phase-based inter-brain connectivity estimates in a large proportion of dyads, such that these estimates may not accurately reflect phase-based coupling across brains."

Minor comments

1. Define "accuracy," "reliability," and "robustness" quantitatively. These terms are used somewhat interchangeably.

We have revised the manuscript to explicitly address this terminology. Specifically, in the Results section, we now state:

"Estimates are referenced to the situation when P1 and P2 share a peak oscillatory frequency of 10 Hz. Here we refer to the accuracy of the connectivity estimates in terms of deviations from this reference value, which corresponds to the maximal connectivity estimate under the present simulation setup. We define robustness as the metric's ability to maintain stable connectivity estimates under perturbations, such as inter-individual frequency differences or variations in signal-to-noise ratio."

We have updated the terminology accordingly throughout the manuscript and removed the use of the term "reliability".

2. Tone in the final paragraph
The phrase about "tempering enthusiasm" is rhetorically strong. It would read as more scientifically grounded if tied directly to the demonstrated limitations (frequency mismatch + metric choice + confounds), rather than as a general statement about "simplified indices."

We have replaced the "tempering enthusiasm" remark with the following statement:

"More broadly, our findings highlight the need to develop or adopt inter-brain connectivity metrics specifically tailored to hyperscanning datasets, and suggest that previous MEG/EEG hyperscanning findings should be interpreted with particular caution due to the sensitivity of commonly-used metrics to frequency mismatch and signal-to-noise ratio."
