# Supplementary material for: Inter-brain functional connectivity: Are we measuring the right thing?
Source: PLoS One. 2026 Jul 14;21(7):e0353371. doi: 10.1371/journal.pone.0353371 (PMC13367725; doi:10.1371/journal.pone.0353371)
Supplement: Peer review history

# Peer review: Decision Letter 0

**DOI:** 10.1371/journal.pone.0353371.r001

**Authors:** Ernesto Pereda

25 Feb 2026

PONE-D-25-64475

Inter-brain functional connectivity: Are we measuring the right thing?

PLOS One

Dear Dr. Avendano-Diaz,

Thank you for submitting your manuscript to PLOS ONE. After careful consideration, we feel that it has merit but does not fully meet PLOS ONE’s publication criteria as it currently stands. Therefore, we invite you to submit a revised version of the manuscript that addresses the points raised during the review process.

Please submit your revised manuscript by Apr 11 2026 11:59PM. If you will need more time than this to complete your revisions, please reply to this message or contact the journal office at plosone@plos.org. When you're ready to submit your revision, log on to https://www.editorialmanager.com/pone/ and select the 'Submissions Needing Revision' folder to locate your manuscript file.

Please include the following items when submitting your revised manuscript:

- A letter that responds to each point raised by the academic editor and reviewer(s). You should upload this letter as a separate file labeled 'Response to Reviewers'.
- A marked-up copy of your manuscript that highlights changes made to the original version. You should upload this as a separate file labeled 'Revised Manuscript with Track Changes'.
- An unmarked version of your revised paper without tracked changes. You should upload this as a separate file labeled 'Manuscript'.

If you would like to make changes to your financial disclosure, please include your updated statement in your cover letter. Guidelines for resubmitting your figure files are available below the reviewer comments at the end of this letter.

If applicable, we recommend that you deposit your laboratory protocols in protocols.io to enhance the reproducibility of your results. Protocols.io assigns your protocol its own identifier (DOI) so that it can be cited independently in the future. For instructions see: https://journals.plos.org/plosone/s/submission-guidelines#loc-laboratory-protocols. Additionally, PLOS ONE offers an option for publishing peer-reviewed Lab Protocol articles, which describe protocols hosted on protocols.io. Read more information on sharing protocols at https://plos.org/protocols?utm_medium=editorial-email&utm_source=authorletters&utm_campaign=protocols.

We look forward to receiving your revised manuscript.

Kind regards,

Ernesto Pereda

Academic Editor

PLOS One

Journal Requirements:

When submitting your revision, we need you to address these additional requirements.

1.Please ensure that your manuscript meets PLOS ONE's style requirements, including those for file naming. The PLOS ONE style templates can be found at

https://journals.plos.org/plosone/s/file?id=wjVg/PLOSOne_formatting_sample_main_body.pdf and

https://journals.plos.org/plosone/s/file?id=ba62/PLOSOne_formatting_sample_title_authors_affiliations.pdf

2. Please note that PLOS One has specific guidelines on code sharing for submissions in which author-generated code underpins the findings in the manuscript. In these cases, we expect all author-generated code to be made available without restrictions upon publication of the work. Please review our guidelines at https://journals.plos.org/plosone/s/materials-and-software-sharing#loc-sharing-code and ensure that your code is shared in a way that follows best practice and facilitates reproducibility and reuse.

3. Please note that funding information should not appear in any section or other areas of your manuscript. We will only publish funding information present in the Funding Statement section of the online submission form. Please remove any funding-related text from the manuscript.

4. Thank you for stating the following financial disclosure:

“J.C.A-D. and L.P. were supported by Business Finland (grant “DIGIMIND” 7981/31/2022). J.C.A-D., R.H., and L.P. were supported by the Norman Loveless Memorial Fund.”

Please state what role the funders took in the study.  If the funders had no role, please state: "The funders had no role in study design, data collection and analysis, decision to publish, or preparation of the manuscript."

If this statement is not correct you must amend it as needed.

Please include this amended Role of Funder statement in your cover letter; we will change the online submission form on your behalf.

5. Thank you for stating the following in the Competing Interests section:

“The authors declare the following competing interests: L.P. has a part-time employment with the MEG device vendor Megin Oy. The other authors declare no competing interests.”

We note that one or more of the authors are employed by a commercial company: MEG device vendor Megin Oy

1. Please provide an amended Funding Statement declaring this commercial affiliation, as well as a statement regarding the Role of Funders in your study. If the funding organization did not play a role in the study design, data collection and analysis, decision to publish, or preparation of the manuscript and only provided financial support in the form of authors' salaries and/or research materials, please review your statements relating to the author contributions, and ensure you have specifically and accurately indicated the role(s) that these authors had in your study. You can update author roles in the Author Contributions section of the online submission form.

Please also include the following statement within your amended Funding Statement.

“The funder provided support in the form of salaries for authors [insert relevant initials], but did not have any additional role in the study design, data collection and analysis, decision to publish, or preparation of the manuscript. The specific roles of these authors are articulated in the ‘author contributions’ section.”

If your commercial affiliation did play a role in your study, please state and explain this role within your updated Funding Statement.

2. Please also provide an updated Competing Interests Statement declaring this commercial affiliation along with any other relevant declarations relating to employment, consultancy, patents, products in development, or marketed products, etc.

Within your Competing Interests Statement, please confirm that this commercial affiliation does not alter your adherence to all PLOS ONE policies on sharing data and materials by including the following statement: "This does not alter our adherence to  PLOS ONE policies on sharing data and materials.” (as detailed online in our guide for authors http://journals.plos.org/plosone/s/competing-interests) . If this adherence statement is not accurate and  there are restrictions on sharing of data and/or materials, please state these. Please note that we cannot proceed with consideration of your article until this information has been declared.

Please include both an updated Funding Statement and Competing Interests Statement in your cover letter. We will change the online submission form on your behalf.

6. Please note that your Data Availability Statement is currently missing the repository name. If your manuscript is accepted for publication, you will be asked to provide these details on a very short timeline. We therefore suggest that you provide this information now, though we will not hold up the peer review process if you are unable.

7. We note that Figure 1 in your submission contain copyrighted images. All PLOS content is published under the Creative Commons Attribution License (CC BY 4.0), which means that the manuscript, images, and Supporting Information files will be freely available online, and any third party is permitted to access, download, copy, distribute, and use these materials in any way, even commercially, with proper attribution. For more information, see our copyright guidelines: http://journals.plos.org/plosone/s/licenses-and-copyright.

We require you to either (1) present written permission from the copyright holder to publish these figures specifically under the CC BY 4.0 license, or (2) remove the figures from your submission:

1. You may seek permission from the original copyright holder of Figure 1 to publish the content specifically under the CC BY 4.0 license.

We recommend that you contact the original copyright holder with the Content Permission Form (http://journals.plos.org/plosone/s/file?id=7c09/content-permission-form.pdf) and the following text:

“I request permission for the open-access journal PLOS ONE to publish XXX under the Creative Commons Attribution License (CCAL) CC BY 4.0 (http://creativecommons.org/licenses/by/4.0/). Please be aware that this license allows unrestricted use and distribution, even commercially, by third parties. Please reply and provide explicit written permission to publish XXX under a CC BY license and complete the attached form.”

Please upload the completed Content Permission Form or other proof of granted permissions as an "Other" file with your submission.

In the figure caption of the copyrighted figure, please include the following text: “Reprinted from [ref] under a CC BY license, with permission from [name of publisher], original copyright [original copyright year].”

2. If you are unable to obtain permission from the original copyright holder to publish these figures under the CC BY 4.0 license or if the copyright holder’s requirements are incompatible with the CC BY 4.0 license, please either i) remove the figure or ii) supply a replacement figure that complies with the CC BY 4.0 license. Please check copyright information on all replacement figures and update the figure caption with source information. If applicable, please specify in the figure caption text when a figure is similar but not identical to the original image and is therefore for illustrative purposes only.

8. If the reviewer comments include a recommendation to cite specific previously published works, please review and evaluate these publications to determine whether they are relevant and should be cited. There is no requirement to cite these works unless the editor has indicated otherwise.

Additional Editor Comments:

Dear authors

Attached are the reviews of your manuscript. As you will notice, particularly in the feedback from Reviewer 1, there are several significant issues that need to be addressed or clarified before your manuscript can be considered for publication again. Please ensure that you address these points while preparing the revised version if you wish to resubmit your manuscript for consideration.

[Note: HTML markup is below. Please do not edit.]

Reviewers' comments:

Reviewer's Responses to Questions

**Comments to the Author**

1. Is the manuscript technically sound, and do the data support the conclusions?

The manuscript must describe a technically sound piece of scientific research with data that supports the conclusions. Experiments must have been conducted rigorously, with appropriate controls, replication, and sample sizes. The conclusions must be drawn appropriately based on the data presented.

Reviewer #1: Yes

Reviewer #2: Partly

**********

2. Has the statistical analysis been performed appropriately and rigorously?

Reviewer #1: Yes

Reviewer #2: Yes

**********

3. Have the authors made all data underlying the findings in their manuscript fully available?

The PLOS Data policy requires authors to make all data underlying the findings described in their manuscript fully available without restriction, with rare exception (please refer to the Data Availability Statement in the manuscript PDF file). The data should be provided as part of the manuscript or its supporting information, or deposited to a public repository. For example, in addition to summary statistics, the data points behind means, medians and variance measures should be available. If there are restrictions on publicly sharing data—e.g. participant privacy or use of data from a third party—those must be specified.

Reviewer #1: Yes

Reviewer #2: No

**********

4. Is the manuscript presented in an intelligible fashion and written in standard English?

PLOS ONE does not copyedit accepted manuscripts, so the language in submitted articles must be clear, correct, and unambiguous. Any typographical or grammatical errors should be corrected at revision, so please note any specific errors here.

Reviewer #1: Yes

Reviewer #2: Yes

**********

5. Review Comments to the Author

Please use the space provided to explain your answers to the questions above. You may also include additional comments for the author, including concerns about dual publication, research ethics, or publication ethics. (Please upload your review as an attachment if it exceeds 20,000 characters)

Reviewer #1: In this ms. the authors describe a much needed and timely study, in which they have used simulations of MEG and EEG data to highlight the need of developing novel and robust connectivity measures for use in inter-/cross-brain analyses of interacting dyads. They start out by reviewing the state of the art, the growing numbers of hyperscanning studies and group studies by the type of connectivity metric used. The authors cogently argue that previous studies have not been able to comprehensively account for inter-individual frequency differences (both across and within individual brains), which explains the need for methodological developments to address this issue. In their simulations they used oscillatory signals with a fixed for one interaction partner and with varying frequency for the other person, while employing various measures of cross-brain functional connectivity in the alpha band. These analyses were repeated for variable SNRs. Results demonstrate that phase-based interbrain connectivity measures were highly sensitive to inter-individual peak frequency differences. In contrast to this, amplitude-envelope-based measures of inter-brain connectivity were more robust to such differences and provide a more reliable measure of amplitude coupling. SNR reductions decreased connectivity estimates for all measures. I only have minor comments and strongly support the publication of this well-written ms. that is likely to make strong contribution to the relevant literature.

-Would it not make sense to also use sets of oscillatory signals with varying frequency for both interaction partners? In recent years, the social interaction mismatch hypothesis as been formulated (e.g. Redcay & Schilbach 2019) and describes the idea that interpersonal similarity - at different levels of scientific assessment - could be related to communication success. Could the here described simulations be extended to address this?

-The authors note that their MEG/EEG signal simulations as continuous sinusoidal oscillations modulated by an identical aperiodic signal that is unlikely to occur in real life, but do not go into any detail how this could be changed or addressed in future research. I would find this quite helpful.

-In the discussion the authors suggest that connectivity metrics should take human physiology into account, but leave open how this could be done. Are thinking more in terms of brain physiology or would they also consider whole-body physiology, as for instance suggested by the growing field of brain-heart-introspection science?

-The authors also stress that cross-brain connectivity measures should be related to social behavior, but also do not touch upon recent developments towards observer-dependent measures of social interaction (e.g. Lahnakoski et al. 2020 and others) that might be helpful along the way.

Reviewer #2: This manuscript uses simulations to examine a well-known property of same-frequency phase-based synchrony metrics in the specific context of MEG/EEG hyperscanning: when two signals have different dominant (peak) frequencies, phase-based measures computed within a fixed band can rapidly lose apparent synchrony. In the presented simulations, PLV and coherence decrease as the peak frequency of participant 2 diverges from participant 1, while amplitude envelope correlation (AEC) remains relatively stable. The question is relevant to hyperscanning practice because inter-individual peak-frequency variability is common, and many studies still apply within-brain connectivity measures to across-brain data without explicit frequency alignment.

However, several aspects of the simulation design and the framing of conclusions make the paper’s claims broader than what is demonstrated. In particular, the simulations appear to favour AEC by construction (shared envelope modulator across participants) and do not establish “ground truth” coupling conditions needed to support strong claims about reliability or about the extent of misestimation in the literature. In addition, novelty and positioning relative to prior methodological discussions are unclear.

Major comments

1. The central effect demonstrated—that phase synchronization metrics at a given frequency degrade when the compared signals are at different frequencies—is a general property of same-frequency phase metrics, not something unique to inter-brain analyses. The manuscript frames the issue as a “critical limitation” of hyperscanning work, but the novelty is currently unclear unless the authors show what is specifically different (or more severe) in hyperscanning practice versus within-brain connectivity (e.g., typical cross-person mismatch distributions, developmental dyads, region-specific peak differences, time-varying peak frequency, etc.). As written, the work risks restating a general signal-processing property without sufficiently demonstrating new insight specific to hyperscanning.

2. Based on the Methods, P1 and P2 signals appear to be generated independently except for sharing an identical slow aperiodic modulator (the oscillation is “amplitude-modulated by an identical aperiodic 1/fχ signal”). This design choice is important: it makes amplitude envelopes correlated across participants by construction, which will favour AEC even when carrier frequencies differ. The Discussion acknowledges that the AEC scenario is idealized, but the main take-home message still implies AEC is broadly “more reliable.”

To support claims about “reliable connectivity estimates,” the simulations should include explicit ground-truth conditions that separate:

• true inter-brain phase coupling (present vs absent),

• true inter-brain amplitude coupling (present vs absent),

• common-driver scenarios (shared exogenous modulation), and

• correlated noise across participants versus independent noise.

Without these controls, the current AEC result is not an informative benchmark for real hyperscanning datasets, where stimulus-locked co-modulation and shared environmental noise are common confounds.

3. Only PLV and “coherence” are used as phase-based measures. Hyperscanning papers commonly use phase-lag metrics aimed at mitigating spurious zero-lag coupling and mixing artefacts (e.g., imaginary coherence, wPLI). Even if these metrics are still sensitive to frequency mismatch, readers will expect them to be considered or clearly explained as out of scope. At minimum, the authors should either (a) include representative phase-lag metrics to show that mismatch sensitivity generalises, or (b) explicitly restrict the scope to frequency-mismatch effects and avoid general claims about “phase-based metrics” in hyperscanning.

Similarly, AEC is often computed with additional steps (e.g., orthogonalization) in EEG/MEG connectivity work to reduce leakage-driven correlations. Because the manuscript raises signal mixing as relevant (especially for EEG), the choice of AEC implementation and its implications should be discussed and/or tested.

4. Band definition and common alignment strategies are not tested

The simulation computes connectivity in a fixed alpha band (7–13 Hz) while P1 is fixed at 10 Hz and P2 varies 6–14 Hz. Many analysis pipelines instead use individual alpha frequency (IAF)-centred bands or compute connectivity at subject-specific peaks. If the practical recommendation is that researchers should not assume identical peak structure across brains, the paper would be more actionable if it tested at least one standard mitigation strategy (e.g., IAF±2 Hz per person, or connectivity computed at each participant’s own peak with an explicit cross-person mapping). As presented, the simulations quantify the expected failure mode of fixed-band, same-frequency phase metrics under mismatch—but do not show how much standard practices mitigate the issue.

5. Claims about the literature are stronger than the evidence provided

The manuscript suggests that many hyperscanning studies “may have misestimated” inter-brain interdependencies due to unaccounted frequency differences. This is plausible, but currently speculative. The paper does not provide evidence about typical mismatch magnitudes in real hyperscanning datasets, nor does it quantify how often common pipelines would produce meaningful underestimation or false negatives. A more defensible statement, based on the simulations shown, would be: phase-based measures computed within a fixed band can strongly underestimate coupling when peak frequencies differ; therefore, studies assuming aligned peaks without testing alignment risk underestimating inter-brain coupling and producing inconsistent results across samples.

Minor comments

1. Define “accuracy,” “reliability,” and “robustness” quantitatively. These terms are used somewhat interchangeably.

2. Tone in the final paragraph

The phrase about “tempering enthusiasm” is rhetorically strong. It would read as more scientifically grounded if tied directly to the demonstrated limitations (frequency mismatch + metric choice + confounds), rather than as a general statement about “simplified indices.”

**********

6. PLOS authors have the option to publish the peer review history of their article (what does this mean?). If published, this will include your full peer review and any attached files.

If you choose “no”, your identity will remain anonymous but your review may still be made public.

**Do you want your identity to be public for this peer review?**  For information about this choice, including consent withdrawal, please see our Privacy Policy.

Reviewer #1: **Yes:** Leonhard Schilbach

Reviewer #2: No

**********

[NOTE: If reviewer comments were submitted as an attachment file, they will be attached to this email and accessible via the submission site. Please log into your account, locate the manuscript record, and check for the action link "View Attachments". If this link does not appear, there are no attachment files.]

To ensure your figures meet our technical requirements, please review our figure guidelines: https://journals.plos.org/plosone/s/figures

You may also use PLOS’s free figure tool, NAAS, to help you prepare publication quality figures: https://journals.plos.org/plosone/s/figures#loc-tools-for-figure-preparation.

NAAS will assess whether your figures meet our technical requirements by comparing each figure against our figure specifications.

# Author comment: Author response to Decision Letter 1

**DOI:** 10.1371/journal.pone.0353371.r002

18 Apr 2026

Uploaded in a separate document

Attachment Submitted filename: Response to Reviewers.pdf

# Peer review: Decision Letter 1

**DOI:** 10.1371/journal.pone.0353371.r003

**Authors:** Ernesto Pereda, Kiyoshi Nakahara

23 Jun 2026

Inter-brain functional connectivity: Are we measuring the right thing?

PONE-D-25-64475R1

Dear Dr. Avendano-Diaz,

We’re pleased to inform you that your manuscript has been judged scientifically suitable for publication and will be formally accepted for publication once it meets all outstanding technical requirements.

Within one week, you’ll receive an e-mail detailing the required amendments. When these have been addressed, you’ll receive a formal acceptance letter and your manuscript will be scheduled for publication.

An invoice will be generated when your article is formally accepted. Please note, if your institution has a publishing partnership with PLOS and your article meets the relevant criteria, all or part of your publication costs will be covered. Please make sure your user information is up-to-date by logging into Editorial Manager at Editorial Manager® and clicking the ‘Update My Information' link at the top of the page. For questions related to billing, please contact billing support.

If your institution or institutions have a press office, please notify them about your upcoming paper to help maximize its impact. If they’ll be preparing press materials, please inform our press team as soon as possible -- no later than 48 hours after receiving the formal acceptance. Your manuscript will remain under strict press embargo until 2 pm Eastern Time on the date of publication. For more information, please contact onepress@plos.org.

Kind regards,

Kiyoshi Nakahara, PhD

Academic Editor

PLOS One

Additional Editor Comments (optional):

Reviewers' comments:

Reviewer's Responses to Questions

**Comments to the Author**

1. If the authors have adequately addressed your comments raised in a previous round of review and you feel that this manuscript is now acceptable for publication, you may indicate that here to bypass the “Comments to the Author” section, enter your conflict of interest statement in the “Confidential to Editor” section, and submit your "Accept" recommendation.

Reviewer #1: All comments have been addressed

**********

2. Is the manuscript technically sound, and do the data support the conclusions?

The manuscript must describe a technically sound piece of scientific research with data that supports the conclusions. Experiments must have been conducted rigorously, with appropriate controls, replication, and sample sizes. The conclusions must be drawn appropriately based on the data presented.

Reviewer #1: (No Response)

**********

3. Has the statistical analysis been performed appropriately and rigorously?

Reviewer #1: (No Response)

**********

4. Have the authors made all data underlying the findings in their manuscript fully available?

The PLOS Data policy requires authors to make all data underlying the findings described in their manuscript fully available without restriction, with rare exception (please refer to the Data Availability Statement in the manuscript PDF file). The data should be provided as part of the manuscript or its supporting information, or deposited to a public repository. For example, in addition to summary statistics, the data points behind means, medians and variance measures should be available. If there are restrictions on publicly sharing data—e.g. participant privacy or use of data from a third party—those must be specified.

Reviewer #1: (No Response)

**********

5. Is the manuscript presented in an intelligible fashion and written in standard English?

PLOS ONE does not copyedit accepted manuscripts, so the language in submitted articles must be clear, correct, and unambiguous. Any typographical or grammatical errors should be corrected at revision, so please note any specific errors here.

Reviewer #1: (No Response)

**********

6. Review Comments to the Author

Please use the space provided to explain your answers to the questions above. You may also include additional comments for the author, including concerns about dual publication, research ethics, or publication ethics. (Please upload your review as an attachment if it exceeds 20,000 characters)

Reviewer #1: (No Response)

**********

7. PLOS authors have the option to publish the peer review history of their article (what does this mean?). If published, this will include your full peer review and any attached files.

If you choose “no”, your identity will remain anonymous but your review may still be made public.

**Do you want your identity to be public for this peer review?**  For information about this choice, including consent withdrawal, please see our Privacy Policy.

Reviewer #1: No

**********
